# Bile acid modulation by gut microbiota: a bridge to understanding cognitive health

**DOI:** 10.1097/MS9.0000000000002433

**Published:** 2024-08-07

**Authors:** Syeda Elezeh Sabahat, Muhammad Saqib, Muneeba Talib, Taha Gul Shaikh, Tooba Khan, Sejal Jain Kailash

**Affiliations:** a Jinnah Sindh Medical University; b Shaheed Mohtarma Benazir Bhutto Medical University, Larkana, Pakistan; c Karachi Medical and Dental College, Karachi, Pakistan; d Dow University of Health Sciences, Karachi; e Vinnytsia National Medical University, Vinnytsia, Ukraine

**Keywords:** bile acids, brain health, cognitive function, gastroenterology, gut microbiome, neurology

## Abstract

The gut microbiota plays an important role in regulating the body’s physiological system, and more recently its impact on bile acid metabolism and cognitive function has been investigated by many studies. In addition to their conventional function in fat digestion and absorption, bile acids are now considered crucial signaling molecules that control several metabolic processes and immunological responses. For this purpose, the authors conducted comprehensive research using relevant terms in an attempt to understand more about the gut microbiota and its impact on bile acid metabolism and cognitive health. The gut-brain axis refers to the network of routes through which gut bacteria communicate with the brain. Through its capacity to bio-transform primary bile acids into secondary bile acids, the gut microbiota plays a significant role in bile acid metabolism. Bile acids function as signaling molecules and act on the brain through nuclear and membrane-bound receptors, influencing neurotransmitter production, neuroinflammation, and neuroplasticity to modify this communication. Any dysregulation in this axis can result in cognitive dysfunction. The link between gut microbiota, bile acids, and cognitive health cannot be ignored. It is imperative to explore this link further by conducting large-scale trials to improve the cognitive health of patients with multiple comorbidities, especially those involving the gastrointestinal tract and nervous system.

## Introduction

Highlights

- The gut microbiome influences important physiological functions and overall well-being of the body.
- Bile acids serve as signaling molecules that regulate metabolic and immunological responses.
- The gut-brain axis is a two-way communication network between gut bacteria and the brain and is influenced by bile acids, affecting neurotransmitter production, neuroinflammation, and neuroplasticity.
- Cognitive dysfunction and neurological disorders are associated with the perturbation of the gut microbiome and bile acid balance.
- Regulating bile acid metabolism and the gut microbiome through diet, probiotics, and prebiotics can potentially assist in the development of therapeutic approaches.

The human microbiome is a complex and dynamic ecosystem of coexisting microorganisms that inhabit the human body^1^. A preponderance of these microbial communities is found within the gastrointestinal tract, extensively in the ileum and colon^2^. An amalgamation of trillions of microbes, including over 100 bacterial species, protozoa, viruses, archaea, fungi, and other biomolecules, constitutes the gut microbiome. These microorganisms colonize the human gut and are pivotal modulators of many crucial bodily functions, influencing their host’s overall well-being. Within the GIT, they have digestive and absorptive activities, participate in the secretion of vitamins such as vitamin K or folate, and form a bulwark against toxins and disease^3,4^. The functional role of gut microbiota is closely related to the physiology and pathology of the human body^5^. It impacts the day-to-day life of its host, with the development and programming of all biological systems being heavily influenced by this relationship. The microbiome crosstalk mediates immunity, homeostasis, brain function, and metabolic pathways^1^.

One such metabolic pathway is that of bile acids. These are a class of water-soluble, amphipathic steroid molecules synthesized via the hepatic catabolism of cholesterol^6^. They are usually conjugated to either taurine or glycine before secretion, which increases their solubility^7^, and the ratio of glycine to taurine conjugates is roughly 3 to 1^8^. Their metabolism involves a chain of reactions, including deconjugation, dehydroxylation, oxidation, epimerization, and re-conjugation by the intestinal microbial population. The importance of bile acids is demonstrated by their pleiotropic effects. Bile acid-sensitive receptors modulate various signaling pathways that influence the metabolic equilibrium of lipids, glucose, steroids, xenobiotics, and energy. They also affect the absorption of intestinal food. This has far-reaching metabolic and immunological implications. Studies have suggested that the physiological importance of bile acids and their signaling may pose promising remedial approaches for metabolic illnesses^9,10^.

The symbiotic association between the gut microbiome and the host has also been shown to influence brain function and cognitive health as early as the first maternal connection in the womb during the embryonic period^11,12^. This interlink is the gut-brain axis (GBA), a two-way network between the gut microflora and the brain. Signaling pathways within the GBA are governed by the gut microbiome, the central nervous system (CNS), and the enteric nervous system (ENS). Communication between microflora and these neural circuits occurs via chemical, hormonal, neuronal, and immunological routes^13^. Neurological disorders are particularly correlated with an imbalance of intestinal bacteria and microbiome diversity. The neural conditions potentially inflicted by gut microbiome perturbation include depression, Parkinson’s disease (PD), Alzheimer’s disease (AD), schizophrenia, multiple sclerosis (MS), and autism spectrum disorders^1^. Moreover, behavioral changes, mood, cognition, overall brain function, development, and inflammation of the nervous system have been observed to be altered as a consequence of dysbiosis^14^. While the gut microbiota’s influence on cognitive function is well-established, its impact extends far beyond the brain. Disruptions in the gut microbiota can contribute to the development of various systemic conditions, including autoimmune disorders like rheumatoid arthritis, lupus, and inflammatory bowel disease^15^, as well as skin conditions like atopic dermatitis and psoriasis^16^. This underscores the critical role of a healthy gut microbiome, not just for cognitive well-being, but for overall bodily function and resilience against a range of disorders.

## Gut microbiome and bile acid metabolism

Roughly 10^12^ microbial organisms inhabit the human gastrointestinal tract, predominantly bacterial species belonging to the *Firmicutes* and *Bacteroidetes* phyla, along with some other phylotypes in a relative minority, that is *Verrucomicrobia, Proteobacteria, Actinobacteria,* and *Fusobacteria* ^17^. This results in a bacterial-to-human cell ratio of 1:1^3^ and 99% of the genomic content in the body being microbial. The composition of microflora colonizing the gut varies from person to person, distinct for each individual^1^. This diversity is brought about by numerous external determinants such as the mode of delivery, dietary habits, breastfeeding, maternal-to-fetal transfer of microbes via vaginal seeding, topographical elements, exposure to drugs, antibiotics, or other environmental toxins, infections, stress, obesity, smoking, and daily physical activity^1,4,9^.

The microbial ecology of the gut plays a pivotal role in the transformation and modification of bile acids and has considerable effects on bile acid toxicity^18^. The bacterial biotransformation of bile acids is heavily regulated by enzymes cultured from gut microbial species^17^. These enzymes participate in bile acid metabolism’s deconjugation, dehydroxylation, and dehydrogenation steps^19^. Dysbiosis of the intestinal microbiome disrupts bile acid metabolism by altering the size and composition of the bile acid pool. Several metabolic illnesses, including inflammatory bowel syndrome (IBS), cholestasis, obesity, gallstone disorders, type 2 diabetes, and nonalcoholic fatty liver disease (NAFLD), result from the microbial manipulation of bile acid signaling^9^.

Bile acids are synthesized via a complex cascade of biochemical reactions involving a minimum of 14 hepatic enzymes^9^. This is attained through either of the two primary biosynthetic pathways, that is the neutral or classic bile acid pathway and the acidic or alternative pathway^20^. In humans, ~200–600 mg of bile acids are synthesized daily. After synthesis, they undergo enterohepatic circulation, are released into bile ducts, and are stored in the gallbladder as a component of bile^10^. Bile acids are then released into the duodenum after food ingestion, where they facilitate the absorption of lipids, TAGs, cholesterol, and lipid-soluble vitamins^19^. This cycle repeats 4–10 times each day, and ~5% of it is eliminated from the body in feces^21^, while a minor proportion remains in the gut each day to function as a substrate for bacteria^18^. These unabsorbed bile acids are metabolized and converted to secondary bile acids by the action of gut microbial species^6^.

The synthesis and circulation of bile acids are kept in order through a closely controlled negative feedback mechanism^19^. As the size of the bile acid pool grows, suppression of its de novo synthesis occurs within the liver and intestine, inhibiting it from piling up and protecting the host from its cytotoxic effects^22^. Additionally, the complex interplay between the four known bile acid-responsive host receptors, primarily the farnesoid X receptor (FXRα), governs bile acid homeostasis^17^. Bile acid levels in systemic circulation are seen to peak postprandially, even more so after the consumption of fatty foods. Similarly, 30 min after an oral glucose tolerance test (OGTT), a rise in the concentration of circulating bile acids is observed^21^. This evidence suggests the involvement of the gut in regulating bile acid homeostasis.

## Gut-brain axis and cognitive function

**Figure 1:**
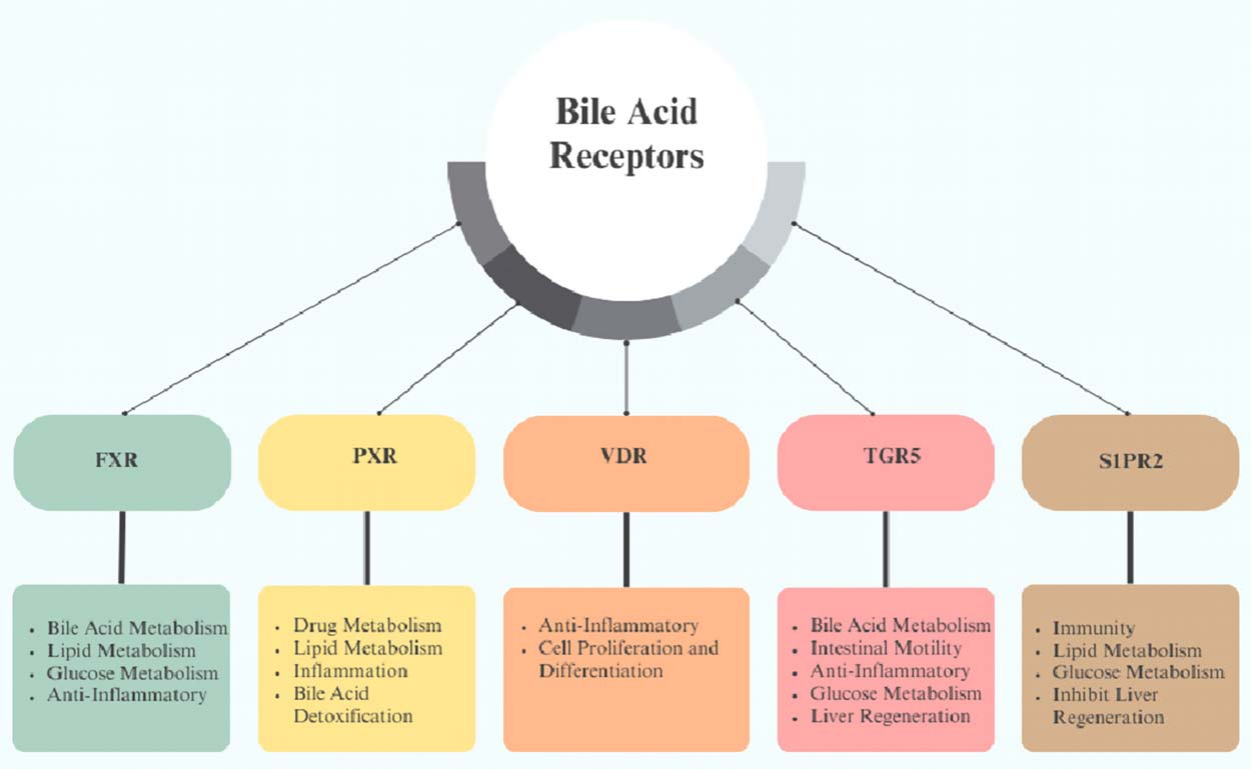
Summary of bile acid receptors and their functions. FXR, farnesoid X receptor; PXR, pregnane X receptor; S1PR2, sphingosine-1-phosphate receptor 2; TGR5, Takeda G protein receptor 5; VDR, vitamin D receptor.

Bile acids may impact how the brain and CNS operate by activating bile acid receptors and altering the gut microbiome^23^. Bile acid signaling occurs through both membrane-bound and nuclear receptors^24^. The farnesoid X receptor (FXR), the pregnane X receptor (PXR), and the vitamin D receptor (VDR) are nuclear receptors, while Sphingosine-1-phosphate receptor 2 (S1PR2), α5β1 integrin, and Takeda G-protein-Coupled receptor 5 (TGR5) present as the membrane-bound receptors. By binding with bile acids, these receptors participate in several physiological processes, including lipid, glucose, and energy metabolism. These receptors serve a commensurate regulatory function by extending the significant physiological responsibilities of bile acids^25^. Both FXR and TGR5 have been detected in the brains of mice and humans^23^. Through feedback inhibition, FXR-mediated bile acid signaling has become one of the main regulators of bile acid production. Recent research has revealed that the FXR receptor is also expressed in the brain, especially in cortical neurons, and that its expression is increased during neuroinflammation, suggesting that illness states might affect the brain’s expression of this receptor. TGR5, along with the liver, lung, and spleen, is also expressed in the brain, in neural cell populations like astrocytes and neurons. TGR5 may have several distinct functions within the CNS and PNS; it reduces neuroinflammation and microglial activation, and it also regulates glucose metabolism, neuronal function, and the immune system. Some reports have shown the expression of sphingosine-1-phosphate receptor (S1PR)2 and α5β1 integrin in the brain. α5β1 integrin interferes with neuronal migration and cortical lamination, influencing brain development. VDR activation in the brain protects from neurodegenerative diseases. However, further studies are required in this association^24^. Figure 1 summarizes the functions of bile acid receptors.

A new realm that has caught the attention of scientists worldwide is the potential link between gut microbiome dysbiosis and cognitive dysfunction^26^. Central to this association appears to be the process of bile acid metabolism. Bile acids control gut bacteria overgrowth, and gut bacteria metabolize bile acids to regulate host metabolism. Alteration of bile acid metabolism by high-fat diets, sleep disruption, alcohol, and drugs reshapes the gut microbiome and causes dysbiosis, obesity, metabolic disorders, and cognitive dysfunction^27^. Gut dysbiosis can lead to chronic inflammation in the gut, which may cause damage to the intestinal barrier and allow harmful substances to leak into the bloodstream. This systemic inflammation can contribute to neurodegenerative disorders like Alzheimer’s and Parkinson’s diseases.

In a research study, the results found that compared to cognitively healthy older individuals, Alzheimer’s disease (AD) patients displayed significantly decreased levels of primary bile acid (BA), namely cholic acid (CA). Conversely, elevated levels of the bacterially derived secondary bile acids, deoxycholic acid, and its glycine and taurine conjugated variants. A higher deoxycholic acid to CA ratio, indicative of 7α-dihydroxylation of CA by gut bacteria, was closely linked to cognitive deterioration. In addition, it was found that several genetic variations in genes related to the immune response, which are involved in AD, had correlations with BA profiles^28^. In a recent clinical investigation, extensive bile acid testing was done on plasma obtained from 30 healthy controls, 20 individuals with mild cognitive impairment, and 30 participants with clinical AD. While levels of glycochenodeoxycholic acid, glycodeoxycholic acid, and glycolithocholic acid were considerably higher in AD patients when compared to patients with mild cognitive impairment, levels of LCA were significantly higher in AD patients when compared to controls. It is unclear how these glycine-conjugated bile acids may be influencing the pathophysiology of AD; however, the presence of LCA and these bile acids show promising biomarker properties for diagnostic reasons. Bile acids were used in other research to modify various pathways of AD pathogenesis, such as enhancing mitochondrial activity or preventing the formation of Aβ. In a rat model of AD neurotoxicity, daily injections of CDCA considerably reduced hippocampus Aβ generation via Aβ42 levels and significantly mitigated AlCl3-induced cognitive and spatial deficits notably similar to the control. The rat model of AD neurotoxicity was established by intraperitoneal injections of AlCl3. Comparing the significant neuronal degeneration in the AlCl3-treated group to the neuroprotective effect of CDCA on the control and CDCA + AlCl3 groups morphologically, hematoxylin and eosin staining indicate.

In conclusion, dynamin-related protein 1 (Drp1) is known to protect against AD-related mitochondrial toxicities. Patients with sporadic and familial AD are linked to mitochondrial damage and morphological abnormalities^29^. Supplementing with some bile acids, such as ursodeoxycholic acid (UDCA) and tauroursodeoxycholic acid (TUDCA), has been shown to decrease neuroinflammation, increase mitochondrial function, and prevent Aβ-induced synaptic toxicity and amyloid precursor protein processing in APP/PS1 mice or neurons^25^.

**Figure 2:**
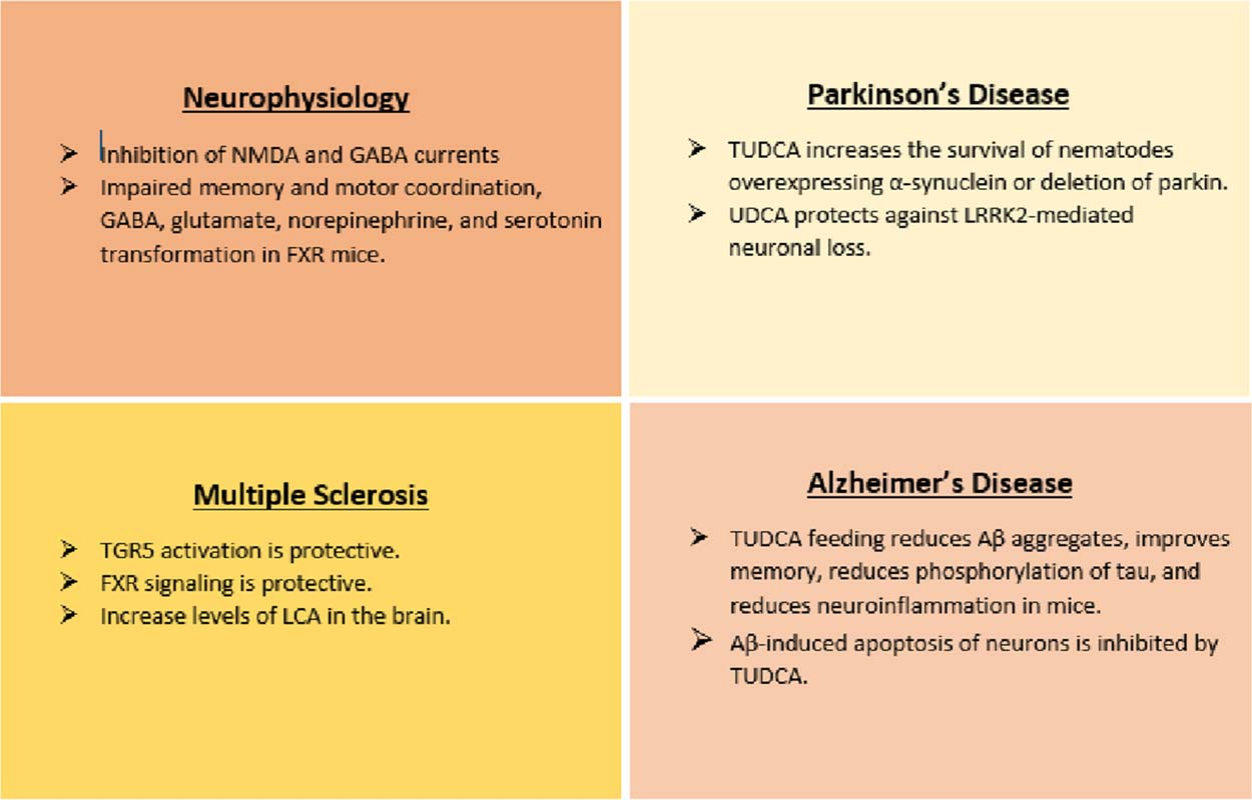
Role of bile acid signaling in neurophysiological and neurodegenerative conditions. FXR, farnesoid X receptor; LRRK2, leucine-rich Repeat Kinase 2; NMDA, N-methyl-D-aspartate; TGR5, Takeda G-protein-coupled receptor 5; TUDCA, tauroursodeoxycholic acid; UDCA, ursodeoxycholic acid.

In Parkinson’s disease, the neuronal loss caused by leucine-rich repeat kinase 2 (LRRK2) G2019S can be reduced by feeding ursodeoxycholic acid (UDCA) via a mechanism involving the restoration of adenosine 5′-triphosphate levels back to normal levels, indicating that this bile acid may be a therapeutic agent for Parkinson’s disease^24^. Likewise, in a recent PD study, TUDCA and UDCA, two anti-inflammatory secondary bile acids, were employed in experimental therapeutic trials. Reduced mitochondrial activity has been linked to PD; the pro-inflammatory cytokine cascade of PD is replicated by the mitochondrial inhibitor 1-methyl-4-phenyl-1,2,3,6-tetrahydropyridine (MPTP). In a mouse model of Parkinson’s disease, a sequence of TUDCA injections was administered both before and after the MPTP injection. Compared to MPTP-treated mice, the motor abilities of the MPTP-treated + TUDCA groups increased, as did their capacity to initiate movements and correct tremors. Parkin levels, an E3 ubiquitin ligase linked to mitochondrial biogenesis, were reduced in mice receiving MPTP treatment and attenuated in animals receiving TUDCA treatment before MPTP treatment^29^. Additionally, it has been discovered that bile acids are intricately linked to several mechanisms in the pathogenesis of hepatic encephalopathy (HE), such as the theory of ammonia intoxication, bile acid circulation, the GABAergic tone hypothesis, and neuroinflammation, which affect patients’ cognitive and motor abilities^30^. Furthermore, in animal models of liver damage, the elevated total bile acid level in brain tissue was discovered before the onset of HE, indicating that bile acid may be an important factor in the pathogenesis of hepatic encephalopathy^25^. Figure 2 highlights some of the roles of bile acid signaling in neurodegenerative diseases and normal physiological processes.

Synaptic plasticity is a crucial neurophysiological phenomenon involved in the formation of brain networks and their reorganization following loss. It describes neurons’ capacity to change the strength of their connections. Another study delved into the impacts of a Western diet (WD), high in fat and sugar, shifts the gut microbiome towards reduced diversity and decreased beneficial bacteria. This imbalance reduces the production of health-promoting short-chain fatty acids (SCFAs) and compromises the gut barrier, allowing harmful bacterial endotoxins into the bloodstream, leading to systemic inflammation, and altering bile acid synthesis. This disruption in bile acid synthesis contributes to systemic inflammation and decreased neuroplasticity, underscoring the integral role of bile acid in the link between a WD, inflammation, and cognitive dysfunction^31^. These pathways underscore the importance of the gut-brain axis and the potential risks associated with gut dysbiosis.

The emerging evidence linking gut microbiome dysbiosis, alterations in bile acid metabolism, and cognitive dysfunction is contributing to a paradigm shift in our understanding of cognitive disorders. It is now apparent that the gut-brain axis is far more complex than initially believed, and maintaining gut health could be a crucial aspect of preserving cognitive function. Future research in this area should aim to elucidate the precise mechanisms behind these interactions and potentially identify therapeutic targets to prevent or slow cognitive decline. A more profound understanding of these complex relationships could pave the way for novel therapeutic strategies, placing the gut at the center of cognitive health interventions.

## Modulation of bile acid metabolism and gut microbiome for brain health

Modulation of bile acid metabolism and the gut microbiome is receiving more attention for its potential impacts on brain health. Different strategies can be used in the modulation of bile acid metabolism and gut microbiome.

Diet has a significant impact on bile acid metabolism and gut microbiome. Certain diets alter the synthesis and secretion of bile acid, which plays an important role in bile acid metabolism. Different studies suggest the impact of dietary patterns in shaping the composition and activity of the gut microbiome, which in turn has a profound effect on bile acid metabolism. For instance, a high-fiber diet is linked to an expansion in the population of gut bacteria, which in turn modulates bile acid metabolism. Fiber is used by gut bacteria as a substrate to produce short-chain fatty acids, which are related to the increased synthesis and secretion of bile acids, thus playing a crucial role in modulating bile acid metabolism. In addition to fiber, A high-fat diet is also linked to an increase in the synthesis of bile acids from cholesterol in the liver^32^. This leads to elevated levels of the bile acid pool for the absorption and digestion of dietary fats. However, it is important to consider the type and quality of dietary fats that can influence bile acid metabolism. The Mediterranean diet, characterized by a high intake of fruits, vegetables, whole grains, and healthy fats, has been linked to healthy bile acid metabolism^33^. The Mediterranean diet has been shown to impact bile acid synthesis and transport. The Mediterranean diet is also rich in fiber, which plays a crucial role in regulating bile acid metabolism, as described above.

Probiotics and prebiotics also play a crucial role in maintaining the healthy homeostasis of the gut microbiome. Probiotics are living microorganisms that have a positive impact on maintaining the normal equilibrium of the gut microbiome. Studies have demonstrated the modulatory effects of probiotics on the gut microbiome, promoting the growth of beneficial bacteria, such as lactic acid bacteria, and inhibiting deleterious ones, like desulfovibrio^34^. Furthermore, probiotics inhibit the growth of pathogens through short-chain fatty acid production and competing for their colonization sites^35,36^. Prebiotics are non-digestible food substances that have been shown to maintain a healthy gut microbiome. Galactooligosaccharides (GOS) and lactulose have been shown to modulate the balance of the gut microbiota significantly. They promote the growth of bifidobacteria and lactic acid bacteria^37^, both bacteria used as probiotics in the treatment of ulcerative colitis and irritable bowel syndrome. Other prebiotics, including pectin, cellulose, and xylan, also play a crucial role in the homeostasis of a healthy gut microbiome^38^.

As previously demonstrated, the crucial role of bile acids in brain health. Therefore, any factor that disturbs the metabolism of bile acid would exert detrimental effects on brain health. Dysregulated bile acid metabolism has been associated with cognitive impairment in diseases such as AD, mild cognitive impairment (MCI), Huntington’s disease (HD), and diabetic cognitive dysfunction (DCD). The exact pathogenesis of each disease state varies. Bile acid-blocking drugs have been shown to increase the risk of dementia. In AD patients, slightly higher levels of cholic acid and chenodeoxycholic acid in postmortem brain tissue were found to be detectable^37^. This suggests the effect of abnormal bile acid metabolism in the progression of AD. In different stages of HD, patients’ levels of bile acid precursors 27-hydroxycholesterol and brain-specific 24S-hydroxycholesterol were observed to be lower than in normal individuals^39^. According to a study, patients with diabetic cognitive dysfunction (DCD) serum levels of glycocholic acid, taurocholic acid, and cholic acid were significantly changed in serum levels of diabetic patients without cognitive impairment and healthy individuals^40^. These findings show the gut microbiota’s influence on dysregulated bile acid signaling, which may contribute to cognitive impairment. However, the underlying mechanisms and potential therapeutic approaches that target bile acid signaling for cognitive impairments are unclear and require further studies to be done.

## Conclusion

To sum it up, this narrative review highlights the impact of the gut microbiome on bile acid metabolism and cognitive function. The gut microbiome has significant importance in bi-directional gut-brain access through its ability to impact the bile acids and neurotransmitters. Furthermore, the relevance of the gut-brain axis in maintaining brain health is highlighted by the bi-directional interaction between the gut microbiome, bile acids, and cognitive performance. The take-home message is that the role of gut microbiota and bile acid metabolism should be considered as key factors in interpreting cognitive performance and brain health. However, several gaps remain in the current understanding. Firstly, many findings in animal models have not yet been validated in human studies, particularly regarding bile acids and their therapeutic potential in neurodegenerative diseases. Mechanistic insights into how gut-derived metabolites influence human brain function are still lacking. Additionally, the impact of gut microbiota dysbiosis on cognitive disorders requires large-scale, longitudinal human studies. The role of genetic variations in shaping gut microbiota and its effects on brain health also warrants further investigation. Further research and clinical trials are required to completely understand the therapeutic implications and create tailored therapies for enhancing the gut microbiome and bile acid metabolism for cognitive well-being.

## Ethical approval

Not applicable.

## Consent

Informed consent was not required since it is a review article.

## Source of funding

This research did not receive any specific grants from the funding agencies.

## Author contribution

S.E.S.: topic idea, literature search, writing, editing. M.S.: literature search, writing. M.T.: literature search, writing. T.G.S.: figure, reviewing, compilation. T.K.: writing and editing. K.S.J.: reviewing and editing.

## Conflicts of interest disclosure

The authors declare no conflict of interest.

## Research registration unique identifying number (UIN)

Not applicable.

## Guarantor

All authors take full responsibility of the work.

## Data availability statement

Not applicable.

## Provenance and peer review

This paper was not invited or commissioned.
